# Targeting cGAS-STING signaling protects retinal ganglion cells from DNA damage-induced cell loss and promotes visual recovery in glaucoma

**DOI:** 10.18632/aging.205900

**Published:** 2024-06-06

**Authors:** Qiuli Zhang, Yinghuan Xiong, Ruizhuang Li, Xiuqin Wang, Xu Lin, Ya’ni Tong

**Affiliations:** 1 Department of Ophthalmology, Affiliated Hospital of Guangdong Medical University, Zhanjiang 524000, Guangdong, China; 2 Biotissue Repository, Affiliated Hospital of Guangdong Medical University, Zhanjiang 524000, Guangdong, China

**Keywords:** glaucoma, cGAS-STING, inflammation, DNA damage, mitochondria lipid peroxidation

## Abstract

Background: Glaucoma is an optic neurodegenerative disease. Retinal ganglion cells (RGCs) are the fundamental neurons in the trabecular meshwork, and their loss is the main pathological reason for glaucoma. The present study was to investigate mechanisms that regulate RGCs survival.

Methods: A mouse model of glaucoma was established by injecting hypertonic saline into the limbal veins. RGCs apoptosis was detected by using flow cytometry. Protein expressions in RGCs in response to DNA damage inducer cisplatin treatment were detected by immunofluorescence and western blot. The expressions of inflammatory cytokines were determined using ELISA and real-time PCR.

Results: In the hypertonic saline-injected mice, we found visual function was impaired followed by the increased expression of γH2AX and activation of cGAS-STING signaling. We found that DNA damage inducer cisplatin treatment incurred significant DNA damage, cell apoptosis, and inflammatory response. Mechanistically, cisplatin treatment triggered activation of the cGAS-STING signaling by disrupting mitochondrial function. Suppression of cGAS-STING ameliorated inflammation and protected visual function in glaucoma mice.

Conclusions: The data demonstrated that cGAS-STING signaling is activated in the damaged retinal ganglion cells, which is associated with increased inflammatory responses, DNA damage, and mitochondrial dysfunction. Targeting the cGAS-STING signaling pathway represents a potential way to alleviate glaucoma-related visual function.

## INTRODUCTION

Glaucoma is a kind of progressive optic neurodegeneration characterized by elevated intraocular pressure (IOP), severe eye pain, and irreversible vision loss that could lead to the progress of permanent blindness [1, 2]. Several risk factors, such as age, genetic factors, thinner corneal thickness, as well as vascular dysregulation, contribute to glaucoma progression [3]. Retinal ganglion cells (RGCs) are the neurons that convey visual information and their loss ultimately causes deficits in neuronal function, which is considered the main pathological hallmark of glaucoma.

The loss of RGCs is triggered by multiple mechanisms, such as neurotrophic factor deprivation, axonal transport failure, activation of apoptotic signals, mitochondrial dysfunction, oxidative stress, and loss of synaptic connectivity, etc. [3]. Ganglion cells are enriched with mitochondria, which control numerous metabolic reactions within the cells, such as oxidative processes [4]. It is now confirmed that cellular injuries induced by aging or ischemia can cause unbalanced oxidative stress in mitochondria by producing uncontrolled levels of ROS, leading to severe cell death [5, 6]. Therefore, oxidation-caused mitochondrial dysfunction is considered a threat to RGCs death. DNA damage is involved in RGCs loss by mediating aging, oxidative stress, post-mitotic neurons, as well as glutamate excitotoxicity, and is considered the major form of neurological disorder [7]. Therefore, strategies that halt and repair DNA damage are recognized to be beneficial for reducing RGCs loss in glaucoma.

DNA damage can be contributed by both endogenous and exogenous factors, such as replication errors, ROS-mediated DNA damage, DNA methylation, ultraviolet light (UV), ionizing radiation (IR), and several chemical agents, that impacts living organisms’ health status [8]. It is believed that DNA damage is regulated by several mechanisms, such as protein modification and signaling pathway dysfunction [8, 9]. Cyclic GMP-AMP (cGAS)-cGAS receptor stimulator of interferon genes (STING) is associated with DNA damage sensing, modulation of inflammatory responses, autoimmunity, and cellular senescence [10–13]. Previous studies showed that inhibition of the cGAS-STING pathway exhibited potential alleviating effects on ischemia/reperfusion injury-induced retinal ganglion cell death [14]. Moreover, diverse effects of the cGAS-STING signaling have been found in mediating ocular diseases including age-related macular degeneration, keratitis, diabetes mellitus, and uveitis [15]. In the present study, we aimed to explore the potential mechanism underlying RGCs loss in glaucoma and the contribution of cGAS/STING signaling to the loss of RGCs in response to DNA stress.

## MATERIALS AND METHODS

### Cell culture

Mouse ganglion cell line RGC-5 cells were purchased from the American Type Culture Collection (ATCC, USA) and maintained in Dulbecco’s Modified Eagle Medium (DMEM) supplemented with 10% FBS in a humidified condition with a mixture of 5% CO_2_ at 37° C.

### Animals and glaucoma model generation

Mice (6-8 weeks) were obtained from The Institute of Laboratory Animals Science of Affiliated Hospital of Guangdong Medical University and maintained under a cycle of 12 hours of light and 12 hours of dark and free access to food and water. Glaucoma model generation was performed according to the previous studies [16, 17]. A total of 30 μL of 2M hypertonic saline was injected into the limbal veins of each mouse and followed by the application of an antibacterial ointment (Neomycin) at the injection site to prevent bacterial infections. IOP was monitored using a tonometer throughout the whole experiment process.

### Visual function measurements

Visual acuity and contrast sensitivity were measured to evaluate the visual function according to previous studies [16, 18]. Briefly, mice were placed on the platform of a chamber surrounded by a computer-assembled camera for 5 minutes before starting measurement. Optomotor responses of each mouse were observed by using an optical microscope (Olympus, Tokyo, Japan). Visual acuity was measured by monitoring the spatial frequency threshold of each animal responding to the rotating grating at a speed of 12°/s. The rotating grating was gradually increased until the mouse no longer exhibited detectable responses. Contrast sensitivity was determined by taking the reciprocal of the contrast threshold until the mouse without any response. The visual function was measured each time a week for 5 weeks.

### Immunofluorescence

The tissue slides were immobilized with 4% PFA and then permeabilized with 1% Triton X-100 for 15 min at room temperature, followed by blocking with 1% BSA for 1 h at room temperature. Slides were incubated with primary antibodies anti-γH2AX (ab81299, Abcam, UK, 1:200 dilution), cGAS (PA5-121188, Invitrogen, USA, 1:100 dilution), STING (90947, Cell Signaling Technology, USA, 1:500 dilution) for overnight. Next, the slides were further incubated with corresponding secondary antibodies Alexa Fluor 488 anti-mouse antibody (4408, Cell Signaling Technology, USA) or Alexa Fluor 555 anti-rabbit antibody (4413, Cell Signaling Technology, USA) for 1 h at room temperature after washing with PBS three times. Then, the slides were stained with DAPI (1:10000) for 10 min and mounted with Gel Mount™ Aqueous Mounting Medium (G0918, Sigma-Aldrich, USA). The slides were visualized under a confocal microscope (Zeiss LSM 800 META, Germany). The average fluorescence intensity per unit area was calculated using image J.

### Western blot

The concentration of cell lysate protein was quantified using a BCA protein assay kit (Thermo Fisher Scientific, USA). Sodium dodecyl sulfate-polyacrylamide gel electrophoresis (SDS-PAGE) was prepared to separate proteins, and the equal amount of protein was then transferred onto polyvinylidene difluoride (PVDF) membranes, which were blocked by 5% BSA for 2 h and incubated with the primary antibodies against cGAS (PA5-121188, Invitrogen, USA 1:1000 dilution), STING (90947, Cell Signaling Technology, USA 1:5000 dilution) at 4° C for 24 h. After washing with PBS 3 times, the membranes were incubated by peroxidase-conjugated secondary antibody (Beyotime, Shanghai, China) for 1 h at room temperature. The bands were visualized by an ECL. The relative values were expressed relative to GAPDH.

### qRT-PCR

Briefly, after being washed with the ice PBS, cells were treated with TRIzol reagent (Invitrogen, USA) to extract total RNA. A Nanodrop Spectrophotometer (Thermo Fisher Scientific, USA) was used to determine RNA purity and concentration. The cDNA was synthesized using Reverse Transcription Kit (Applied Biosystems, USA) according to the manufacturer’s instructions. PCR was performed using a Lightcycler 480 Real-time PCR system (Roche, Mannheim, Germany) with SYBR Green qPCR Master Mix (Thermo Fisher Scientific, USA). The relative expression of mRNA was calculated through the 2^-ΔΔt^ method and normalized to GAPDH. Each experiment was performed at least in triplicate.

### Determination of cytokine levels

Cell supernatant levels of IL-6, IFN-β, and CXCL10 were analyzed by commercially available ELISA sets following the manufacturer’s instructions (R&D Systems, USA).

### Flow cytometry

For cell apoptosis, cells were stained with PI-Annexin V-FITC (Beyotime, China) for 30 minutes at 4° C. Analyses were quantified by using a flow cytometer (BD FACSCantoII; BD Biosciences, USA). For ROS detection, cells were labeled with C11 BODIPY 581/591 (D3861, Invitrogen, USA). The fluorescent signal was quantified by using a flow cytometer (BD FACSCantoII; BD Biosciences, USA). The mitochondrial membrane potential (MMP) was measured using the JC-1 Kit (Sigma-Aldrich, USA). Cells were incubated JC-1 for 30 min. The fluorescence signals were measured using a flow cytometer (BD FACSCantoII; BD Biosciences, USA).

### Mitochondrial function analysis

Briefly, 1.0×10^4^ cells/well were seeded onto each well of an XF96 cell culture microplate. The oxygen consumption ratio (OCR) was assessed in a glucose-containing XF base medium according to the manufacturer’s instructions. For ATP respiration, ATP levels were quantified using The CellTiter-Glo® Luminescent Cell Viability Assay (Promega, USA). Briefly, cells were exposed to CellTiter-Glo reagent at a 96-well plate and then placed on an orbital shaker platform at 200 rpm for 2 min, followed by incubating at room temperature for 10 min. The luminescence was visualized using a Synergy™ 4 Hybrid Microplate Reader (BioTek, USA). ATP production was detected using an ATP synthase kit (ab109714). Briefly, the passaged cells were frozen at -80° C, after thawing, the resuspended cells were treated by the solution 1 media supplied in the assay kit. After being detected by a BCA protein assay, the cells were centrifuged for 20 min at 16,000 RPM, followed by transferring to a 96-well plate of 50 μL with each sample and coated with monoclonal antibodies specific to ATP synthase for 3 h at room temperature. After being washed by solution 1 media, cells were incubated by lipid mix for 45 minutes and then incubated with 200 μL of reagent mix. Finally, cells were visualized at 340 absorbances using a BioTek Synergy H1 Spectrophotometer.

### Statistical analysis

All data are shown as the mean ± s.e.m. The two-tailed t-test was applied to detect statistical differences between the two groups using GraphPad Prism 8 software. P-values less than 0.05 were considered statistically significant.

## RESULTS

### DNA damage is detected in an experimental model of glaucoma

**Figure 1:**
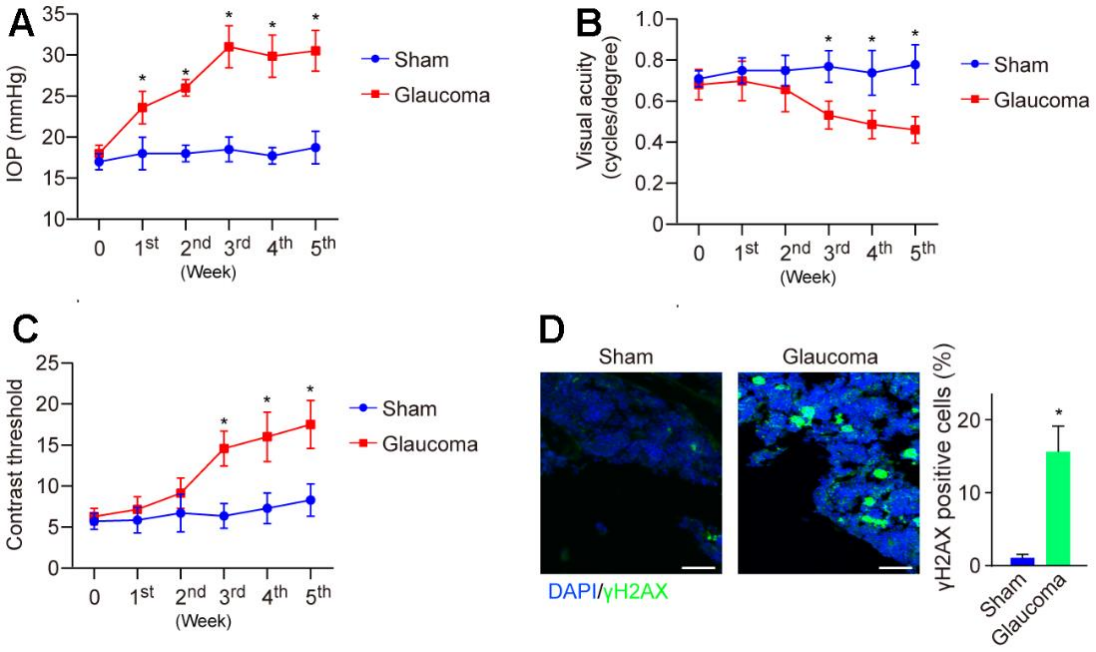
**Visual function and DNA damage in the mice model of HS-induced glaucoma.** (**A**) IOP was dynamically monitored weekly using a tonometer for 5 weeks. (**B**) Visual acuity was measured by monitoring the spatial frequency threshold of each animal responding to the rotating grating at a speed of 12°/s. (**C**) Contrast sensitivity was determined by taking the reciprocal of the contrast threshold until the mouse without any response. (**D**) DNA damage of the mice's retina at the end of the 5^th^ week was detected by immunofluorescence. *p<0.05 vs Sham. n=5 of each group.

Glaucoma is a degenerative disease characterized by the loss of RGCs that can be triggered by multiple factors, such as DNA damage [19]. Here, to study the role of DNA damage in the pathogenesis of glaucoma, we generated an experimental model of glaucoma by using hypertonic saline (HS) injection as reported previously [16]. After injection, IOP was dynamically monitored weekly and showed that IOP was gradually elevated and reached a peak in the 3^rd^ week as compared to the sham group (Figure 1A). Visual function was also impaired (Figure 1B, 1C). Subsequently, the mice were sacrificed at the end of the 5^th^ week, and the retina was collected and subjected to immunofluorescence (IF) to detect DNA damage. We found that the level of γH2AX, a DNA damage marker, was significantly elevated in the glaucoma group compared with the sham group (Figure 1D). The data demonstrated increased DNA damage in glaucoma.

### cGAS-STING mediates DNA damage-dependent RGC apoptosis

**Figure 2:**
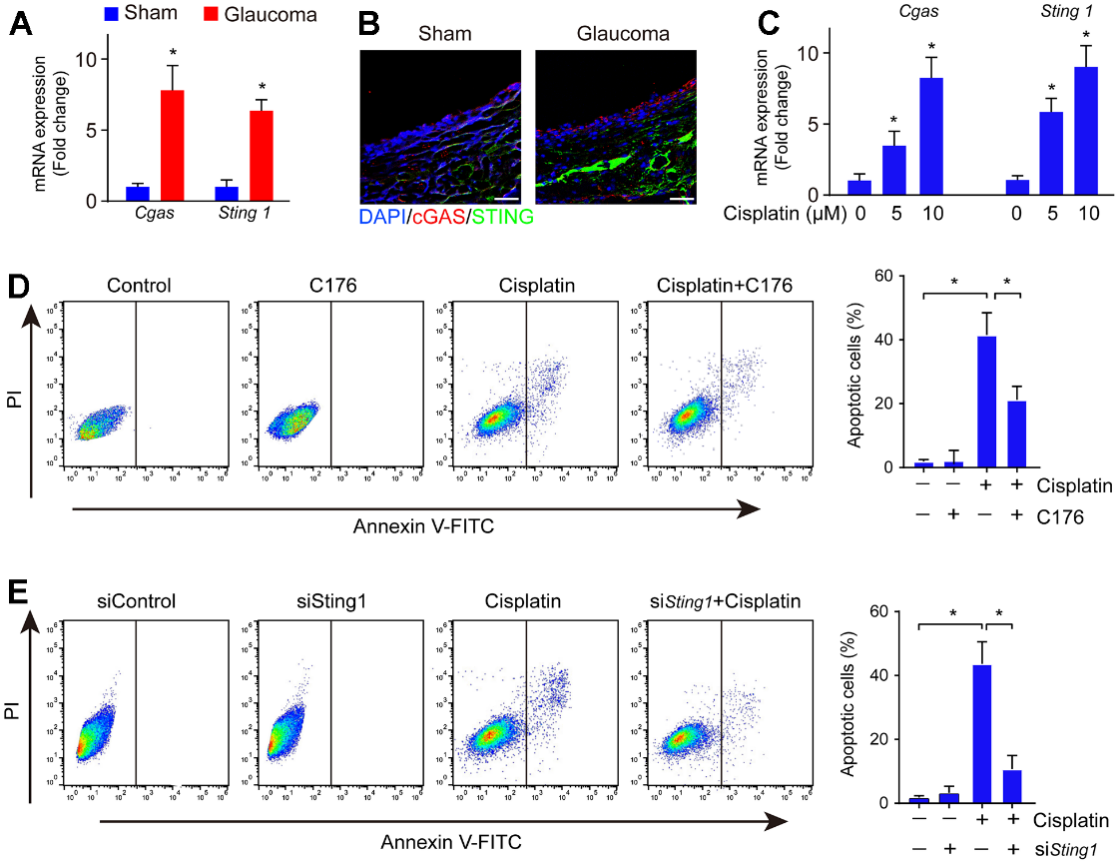
**cGAS-STING activation occurred along with RGC apoptosis in DNA damage.** (**A**) The mRNA expression of cGAS and STING in the glaucoma retina was detected by real-time PCR. (**B**) The expression of cGAS and STING in the glaucoma retina was detected by immunofluorescence. (**C**) The mRNA expression of cGAS and STING in cell line RGC-5 after being treated with cisplatin was detected by real-time PCR. (**D**) Cell apoptosis was detected by flow cytometry after RGC-5 cells were treated with C176 and cisplatin. (**E**) Cell apoptosis was detected by flow cytometry after RGC-5 cells were treated with C176 (siSTING) combined with cisplatin. *p<0.05.

The cGAS-STING pathway implicates DNA damage sensing [20]. To explore whether cGAS-STING signaling is involved in the pathology of glaucoma, we investigated the mRNA expression of cGAS and STING and found that cGAS and STING were significantly upregulated in the glaucoma retina (Figure 2A). Consistently, its protein expression was also highly expressed in the glaucoma group (Figure 2B). Glaucoma is associated with RGC loss leading to eventual visual dysfunction. Next, we investigated if cGAS-STING signaling mediated RGC loss *in vitro*. Firstly, cell line RGC-5 was treated with cisplatin to reassembly induce DNA damage, followed by treatment with STING inhibitor C176 to inhibit cGAS-STING signaling activity. We found treatment with cisplatin induced DNA damage as well as activation of the cGAS-STING signaling in RGC cells (Figure 2C). The addition of cGAS-STING signaling inhibitor C176 significantly reversed cisplatin-induced RGC cell apoptosis (Figure 2D). Moreover, genetic inhibition of STING significantly abrogated cisplatin-induced RGC cell apoptosis (Figure 2E). The data highlight the crucial role of cGAS-STING in regulating DNA damage-induced cell death in RGC.

### Activation of cGAS-STING induces inflammation

**Figure 3:**
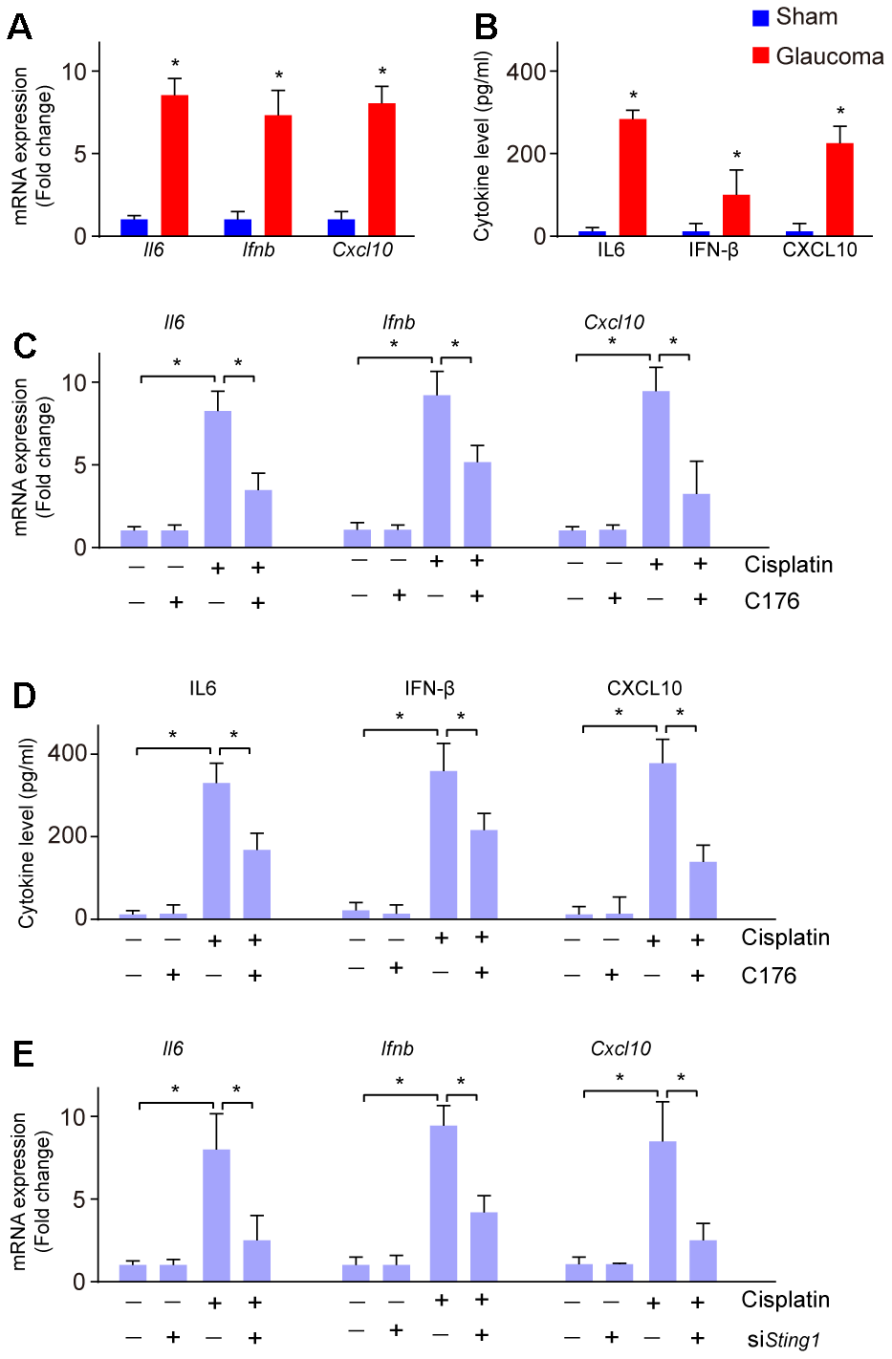
**Inflammatory response to cGAS-STING activation in glaucoma.** (**A**) The mRNA expression of *Il6, Ifnb,* and *Cxcl10* in glaucoma was detected by real-time PCR. (**B**) ELISA analysis was conducted to measure the level of IL-6, IFN-β, and CXCL10 in glaucoma. (**C**) mRNA expression of *Il6, Ifnb,* and *Cxcl10* in RGC-5 cells treated with cisplatin, followed by C176 was detected by real-time PCR. (**D**) The cytokine level of *Il6, Ifnb,* and *Cxcl10* in RGC-5 cells treated with cisplatin, followed by C176 was detected by ELISA. (**E**) Genetic inhibition of cGAS-STING combined with cisplatin on the expression of *Il6, Ifnb,* and *Cxcl10* in RGC-5 cells was detected by real-time PCR. *p<0.05.

Activation of cGAS-STING also leads to the intracellular inflammatory response [21]. Next, we investigated whether the inflammatory response was increased in glaucoma. We found the mRNA expression of cGAS-STING signaling downstream genes *Il6, Ifnb,* and *Cxcl10* were increased in glaucoma as compared to the sham group (Figure 3A). Moreover, ELISA analysis showed the level of IL-6, IFN-β, and CXCL10 were also elevated in glaucoma as compared to the sham group (Figure 3B), suggesting an inflammatory response in glaucoma. To uncover whether the inflammatory response is associated with the activation of cGAS-STING signaling *in vitro*, RGC-5 cells were treated with cisplatin, followed by treatment with C176. C176 significantly restored cisplatin-induced increased mRNA expression of *Il6, Ifnb,* and *Cxcl10* (Figure 3C). Moreover, the amount of the secreted IL-6, IFN-β, and CXCL10 were also repressed by C176 treatment (Figure 3D). Genetic inhibition of cGAS-STING also remarkably reversed cisplatin-induced increased mRNA expression of *Il6, Ifnb,* and *Cxcl10* (Figure 3E).

### Activation of cGAS-STING induced by cisplatin is regulated by lipid oxidation

**Figure 4:**
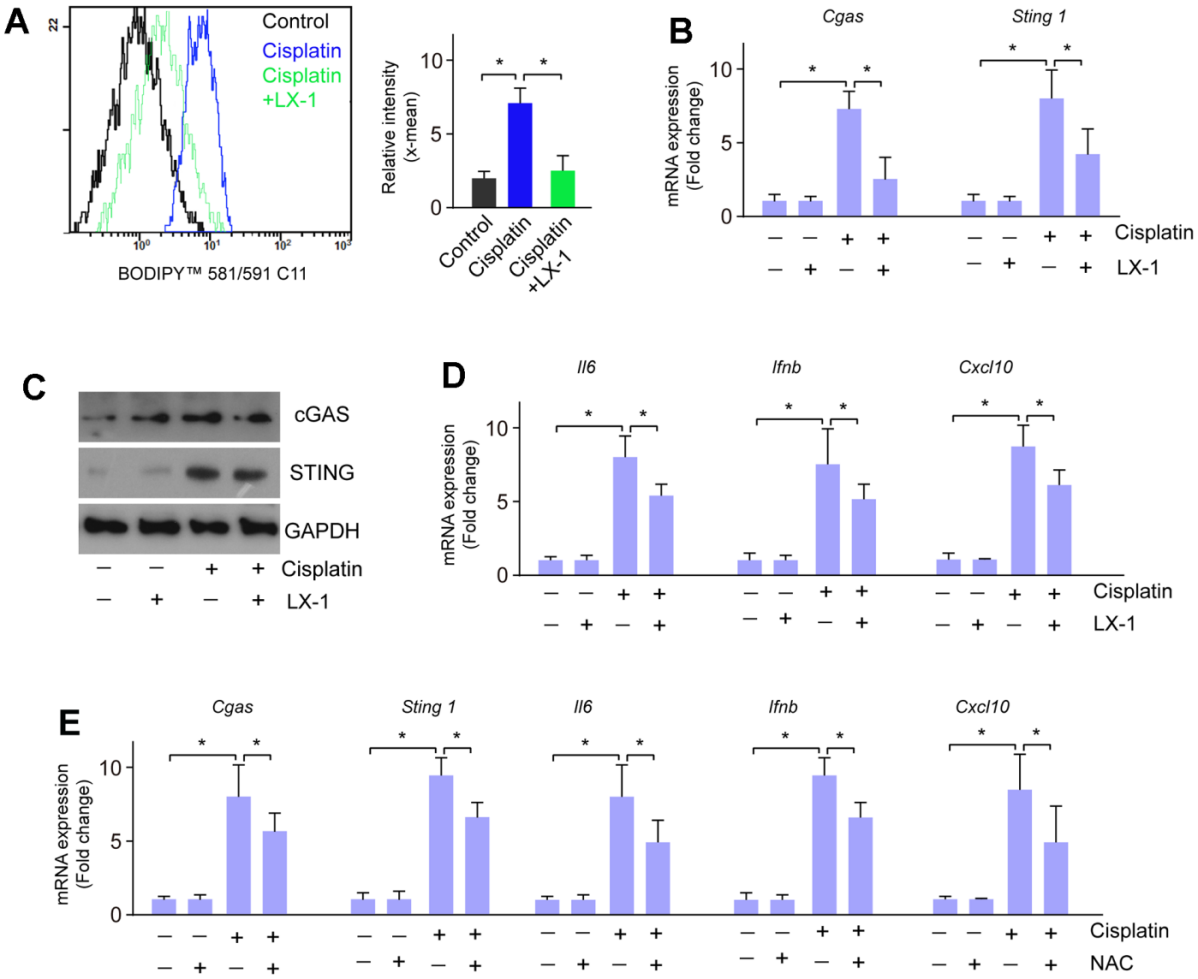
**Lipid oxidation modulates the activation of cGAS-STING induced by cisplatin.** (**A**) Lipid oxidation was detected when RGC-5 cells were treated with cisplatin followed by LX-1. (**B**) mRNA expression of cGAS and STING was detected by real-time PCR when RGC-5 cells were treated with cisplatin followed by LX-1. (**C**) Protein expression of cGAS and STING was detected by western blot when RGC-5 cells were treated with cisplatin followed by LX-1. (**D**) mRNA expression of *Il6, Ifnb,* and *Cxcl10* was detected by real-time PCR when RGC-5 cells were treated with cisplatin followed by LX-1. (**E**) The mRNA expression of cGAS, STING, *Il6, Ifnb,* and *Cxcl10* was detected by real-time PCR in RGC-5 cells that were treated with cisplatin followed by NAC. *p<0.05.

Next, we investigated whether the activation of cGAS-STING induced by cisplatin is regulated through lipid oxidation. RGC-5 cells were treated with cisplatin, followed by treatment with lipid oxidation inhibitor liproxstatin-1 (LX-1), and N-acetyl-l-cysteine (NAC), respectively. Cisplatin treatment induced lipid oxidation, which is markedly restored by LX-1 pretreatment (Figure 4A). LX-1 also alleviated cisplatin-induced activation of cGAS-STING signaling as well as the downstream gene expression (Figure 4B–4D). Similarly, treatment with NAC also reversed restored cisplatin-induced activation of cGAS-STING signaling and downstream gene expression of *Il6, Ifnb,* and *Cxcl10* (Figure 4E). These data imply that lipid peroxidation is linked to cisplatin-induced cGAS-STING activation.

### Lipid oxidation caused by cisplatin through mitochondrial dysfunction

**Figure 5:**
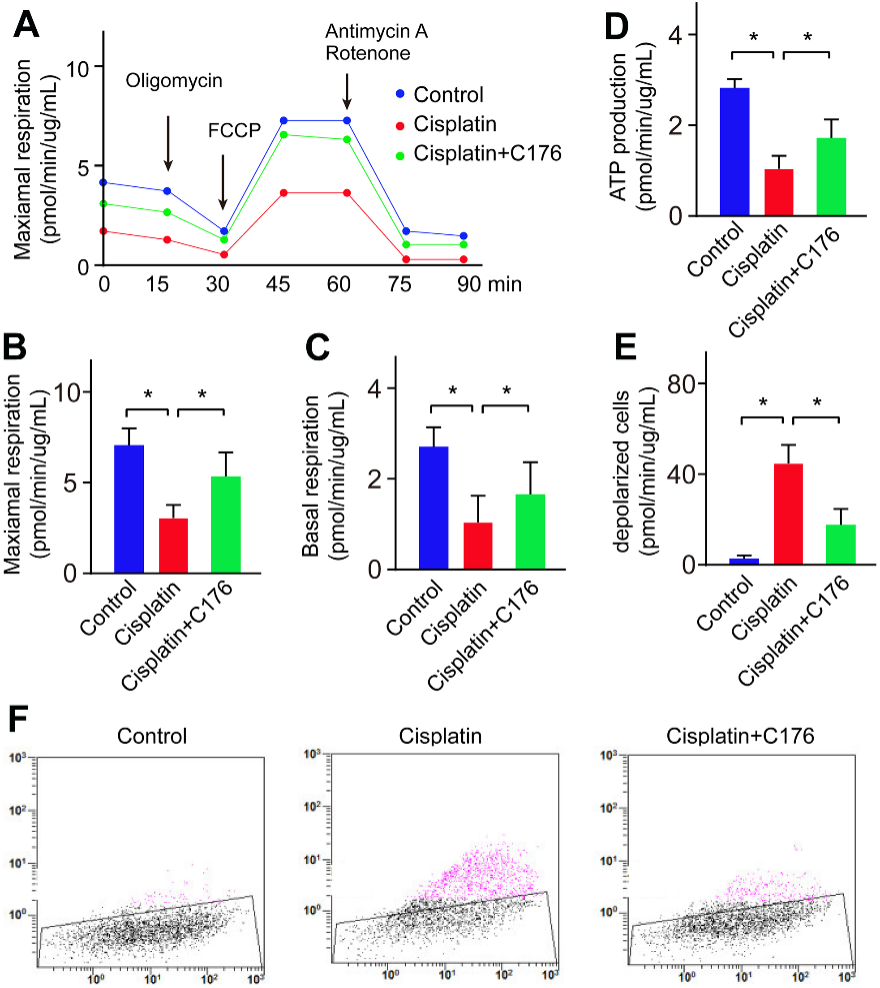
**Cisplatin induces lipid oxidation by causing mitochondrial dysfunction.** (**A**, **B**) Maximal respiration was detected in RGC-5 cells when treated with cisplatin followed by C176. (**C**) Basal respiration was detected in RGC-5 cells when treated with cisplatin followed by C176. (**D**) ATP production was recorded in RGC-5 cells when treated with cisplatin followed by C176. (**E**, **F**) Mitochondrial membrane potential was determined in RGC-5 cells when treated with cisplatin followed by C176. *p<0.05.

It reported that activation of cGAS-STING-induced inflammatory response linked to the mitochondrial functional defect. To address the mechanism by which activation of the cGAS-STING pathway in cisplatin-induced RGC inflammation, we focused on the link between the cGAS-STING pathway and mitochondrial damage. We found cisplatin indeed impaired mitochondrial respiration ability and mitochondrial ATP production (Figure 5A–5D). Next, we investigated the change of mitochondrial membrane potential, as it is depolarized when mitochondrial dysfunction. We found mitochondrial membrane potential was significantly reduced, manifested by the increased percentage of depolarized cells (Figure 5E, 5F). C176 treatment significantly rescued mitochondrial respiration ability, mitochondrial ATP production and mitochondrial membrane potential depolarization (Figure 5A–5F).

### Suppression of cGAS-STING ameliorates inflammation and protects the visual function

**Figure 6:**
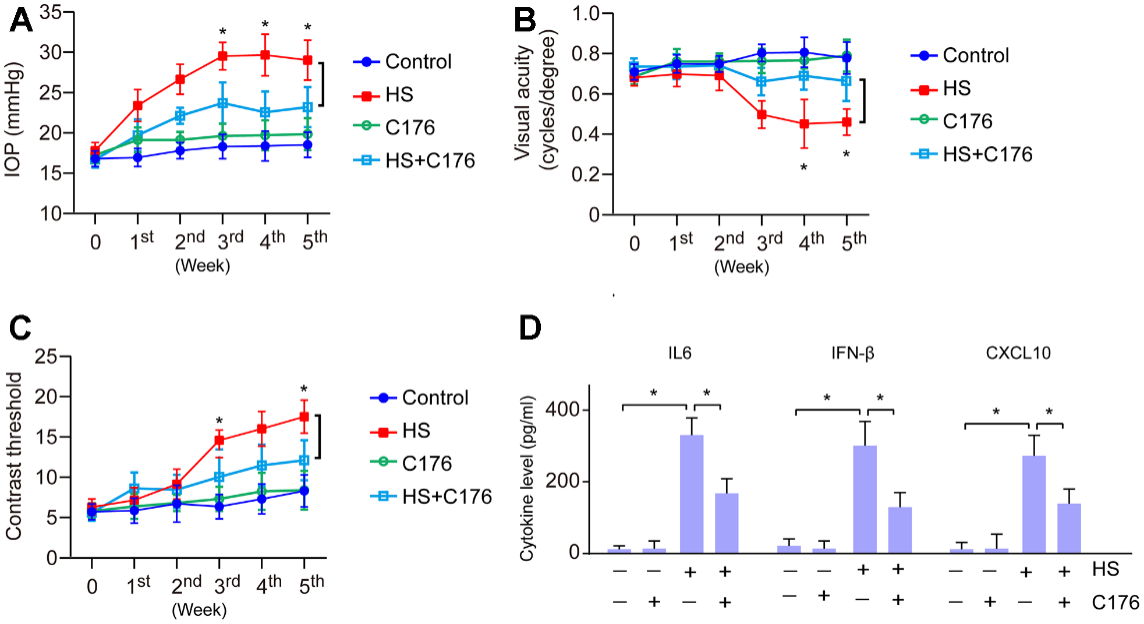
**Role of cGAS-STING on the inflammatory response and visual function.** (**A**) The role of STING inhibitor C176 on IOP of HS-induced glaucoma mice. (**B**) The role of C176 on the visual acuity of HS-induced glaucoma mice. (**C**) The role of C176 on the contrast threshold of HS-induced glaucoma mice. (**D**) The role of C176 on pro-inflammatory molecule expression. *p<0.05.

To confirm the effect of the cGAS-STING pathway on the progression of glaucoma, we utilized STING inhibitor C176 to treat HS-induced glaucoma. We found that C176 treatment exhibited no remarkable effects on normal IOP and visual function, but significantly attenuated HS-induced visual dysfunction and increased IOP as compared to control mice (Figure 6A–6C). We next investigated if this effect was associated with the inflammatory response. We found this alleviating effect was accompanied by a decrease in pro-inflammatory molecule expression (Figure 6D). Our data demonstrate that suppression of cGAS-STING represents a promising way to ameliorate retinal inflammation and protect visual function in glaucoma.

## DISCUSSION

Glaucoma is a heterogeneous group of eye conditions characterized by elevated IOP enlarged cornea and globe and severely induced blindness in 15% of cases worldwide [22]. The degeneration of RGCs is the main reason for glaucomatous visual loss, which leads to visual impairment, while the mechanism that causes RGCs degeneration remains incompletely understood. Early studies have shown that neurodegeneration and atrophy was occurred in glaucoma patients, additionally, the RGCs degeneration also related to endoplasmic reticulum stress, and peroxynitrite-mediated oxidative stress damage [23]. Recently, DNA damage has been observed to result in the loss of RGCs, and the damaged DNA is associated with molecular expression patterns, inflammatory responses, signaling transduction, and activation [24]. Therefore, studying the molecular events under DNA damage in RGCs will provide the theoretical basis for preventing vision loss and improving visual outcomes.

Cisplatin is a common chemotherapeutic drug for various solid tumors, such as ovarian, lung, bladder, cervical, and head and neck neoplasms [25]. Cisplatin causes DNA lesions and further results in cell death by disruption of nuclear DNA, and distortion of the helical structure of the DNA molecule, DNA replication, and transcription [26, 27]. The cyclic GMP-AMP synthase (cGAS) is a cytosolic DNA sensor and plays an important role in microbial pathogens-induced invasion [28]. Activation of cGAS further stimulates STING to trigger interferon signaling [29–31]. Therefore, the cGAS-STING pathway is considered an evolutionary conserved defense mechanism against viral infections. In the present study, cisplatin was used to induce DNA damage in RGCs, followed by the increased activation of cGAS-STING. While cGAS-STING signaling inhibitor C176 significantly abrogated cisplatin-induced RGCs cell apoptosis, indicating a crucial role of cGAS-STING in regulating cisplatin caused DNA damage in RGCs. Besides the diverse roles in microbial infections, cGAS-STING signaling was found in multiple pathological processes, such as cell death, tumors, as well as inflammation [32–34]. Additionally, mitochondrial DNA damage causes inflammation by activation of cGAS-STING signaling in acute kidney injury [34]. Further, the cGAS-STING signaling pathway also emerged as a key mediator in inflammation and regulating infection, cellular stress as well as tissue damage [35]. In the present study, we found that cGAS-STING is activated in the glaucoma retina of the hypertonic saline-induced mouse model. Further studies identified that the activated cGAS-STING is related to cell death and the inflammatory response of RGCs. All those results demonstrated that cisplatin caused RGC damage by activating the cGAS-STING signaling pathway.

Next, we explored the potential mechanism of cisplatin activating cGAS-STING signaling. Although it is well known for DNA damage, cisplatin also causes cytoplasmic organelle dysfunction, particularly with the endoplasmic reticulum and mitochondria [36]. Cisplatin is reported to induce mitochondrial injury by changing mitochondrial morphology, reducing mitochondrial DNA content, and altering mitochondrial gene expression [37]. Lipids are essential for physiological processes by maintaining membrane integrity, providing a source of energy acting as signaling molecules, and further controlling normal cell proliferation, metabolism, inflammation, and apoptosis [38]. It has been reported that the dysfunction of mitochondria is partly mediated by lipid peroxidation, and further induces DNA damage [38]. Herein, consistent with the previous research, the present study showed that cisplatin treatment impaired mitochondrial respiration ability and mitochondrial ATP production, mitochondrial membrane potential was significantly reduced, and the depolarized cells were increased. All those results indicated that cisplatin induced lipid oxidation through mitochondrial dysfunction, and further linked to the cisplatin-induced cGAS-STING activation.

Taken together, this study presented that the glaucoma retina showed significant DNA damage along with the activation of cGAS-STING, which is followed by cell death, inflammation, as well as mitochondria dysfunction-related lipid oxidation. The cGAS-STING signaling represents a potential therapeutic strategy for glaucoma.
